# Colorectal cancer in Lynch syndrome families: consequences of gene germline mutations and the gut microbiota

**DOI:** 10.1186/s13023-025-03543-4

**Published:** 2025-01-18

**Authors:** Xuexin Wang, Zhijun Zheng, Dongliang Yu, Xiaojue Qiu, Ting Yang, Ruoran Li, Jing Liu, Xin Wang, Peng Jin, Jianqiu Sheng, Nan Qin, Na Li, Junfeng Xu

**Affiliations:** 1 Medical School of Chinese PLA, Beijing, 100853 China; 2 Realbio Genomics Institute, Shanghai, 201114 China; 3 Department of Gastroenterology, The Seventh Medical Center of Chinese PLA General Hospital, Beijing, 100700 China; 4 Senior Department of Gastroenterology, The First Medical Center of Chinese PLA General Hospital, Beijing, 100853 China; 5 Tenth People’s Hospital of Tongji University, Shanghai, 200072 China; 6 Qingdao Realbio Precision Medical Test Co. Ltd, Qingdao, 266071 China

**Keywords:** Lynch syndrome, Colorectal cancer, Gut microbiota, 16S rRNA gene amplicon sequencing, Germline gene mutation, Butyrate-producing bacteria

## Abstract

**Background:**

Lynch syndrome (LS)-associated colorectal cancer (CRC) always ascribes to pathogenic germline mutations in mismatch repair (MMR) genes. However, the penetrance of CRC varies among those with the same MMR gene mutation. Thus, we hypothesized that the gut microbiota is also involved in CRC development in LS families.

**Methods:**

This prospective, observational study was performed from December 2020 to March 2023. We enrolled 72 individuals from 9 LS families across six provinces in China and employed 16S rRNA gene amplicon sequencing to analyze the fecal microbiota components among LS-related CRC patients (AS group), their spouses (BS group), mutation carriers without CRC (CS group), and non-mutation carriers (DS group) using alpha and beta diversity indices.

**Results:**

There were no apparent differences in age or gender among the four groups. Alpha and beta diversity indices exhibited no significant differences between the AS and BS groups, verifying the role of germline mutations in the occurrence of CRC in LS families. Beta diversity analysis exhibited significant differences between the AS and CS groups, revealing the importance of the gut microbiota for the occurrence of CRC in LS families. A greater difference (both alpha and beta diversity indices) was shown between the AS and DS groups, demonstrating the combined impact of the gut microbiota and genetic germline mutations on the occurrence of CRC in LS families. Compared with those in the CS and DS groups, we identified ten microbial genera enriched in the AS group, and one genus (*Bacteroide*s) decreased in the AS group. Among the elevated genera in the AS group, *Agathobacter*, *Coprococcus* and *Prevotellaceae_NK3B31_group* were butyrate-producing genera.

**Conclusion:**

This study found the development of CRC in the LS families can be attributed to the combined effects of gene germline mutations as well as the gut microbiota and provided novel insights into the prevention and treatment of CRC in the LS families.

**Supplementary Information:**

The online version contains supplementary material available at 10.1186/s13023-025-03543-4.

## Background

Colorectal cancer (CRC) is the second most common malignancy in females (9.4%) and the third most common malignancy in males (10.6%) [1]. Concerning the genesis of the mutation, CRC can be categorized as sporadic (70%), hereditary (5%), and familial (25%) [2]. Hereditary CRC comprises polyposis forms, such as familial adenomatous polyposis, and nonpolyposis forms, such as Lynch syndrome (LS). Lynch syndrome, formerly referred to as hereditary nonpolyposis colorectal cancer syndrome, is the most common genetic syndrome and accounts for approximately 3% of unselected patients with CRC [3]. Traditionally, the propensity of LS families for CRC or other cancers can be attributed to the presence of pathogenic mutations in mismatch repair (MMR) genes (MLH1, MSH2, MSH6, PMS2, or PMS1) or a germline deletion in EPCAM that eventually results in the epigenetic silencing of the MSH2 gene [4, 5].

MMR proteins function to correct DNA base pair mismatches during DNA replication and recombination. Loss of MMR proteins triggers somatic hypermutation in cancer-associated genes with coding DNA repeats [6]. The most commonly mutated MMR genes are MLH1 and MSH2, followed by MSH6 and, less frequently, PMS2 [6]. Loss of the MLH1 or MSH2 protein in tumors always leads to the concurrent loss of PMS2 or MSH6, respectively. However, a mutation in PMS2 or MSH6 does not typically result in the concurrent loss of MLH1 or MSH2 in the tumor, likely due to PMS1 or MLH3 replacing PMS2 and MSH3 replacing MSH6 [6]. The type of affected MMR gene impacts the CRC incidence. The cumulative risk of CRC at 75 years is 46%, 43%, and 15% for path_MLH1, path_MSH2, and path_MSH6 carriers, respectively, while the cumulative incidence of CRC at 80 years is 13% for men and 12% for women among PMS2 mutation carriers [7, 8].

In addition to genetic predispositions, environmental factors, especially the gut microbiota, play an integral role in CRC development and progression [9, 10]. The gut microbiota converts mutant p53 from tumor-suppressive to oncogenic in mice models of intestinal cancer [11]. Several studies have been conducted on the correlation between microbiota composition and sporadic CRC [12]. Substantial discrepancies have been reported between the gut microbiota of CRC patients and healthy individuals [13]. However, little is known about the function of the gut microbiota in hereditary CRC. Animal experiments have revealed that the microbiota composition affects the incidence of mutagenesis in MSH2-deficient crypts [14]. A recent study revealed that the penetrance of CRC differs among those with the same MMR gene mutation, specifically among MLH1 and MSH2 mutation carriers [15]. Furthermore, we discovered that LS family individuals with MMR mutations do not always develop CRC or other cancer types. Thus, these gene germline mutations cannot fully explain the development of CRC in LS families.

Therefore, we aimed to investigate and elucidate the role of the gut microbiota and genetic germline mutations in the onset of CRC in LS families. We hypothesized that CRC in LS families may be attributable to the combination of the gut microbiome and genetics.

## Methods

### Patients and samples collection

Participants were enrolled from 9 LS families across six provinces in China from December 2020 to March 2023 at the Seventh Medical Center of PLA General Hospital. The inclusion criteria were as follows: (1) a diagnosis of LS consistent with the Amsterdam II criteria [16]; (2) fecal samples collected before colonoscopy or at least 2 weeks interval with colonoscopy; (3) no antibiotics or other drug use for at least 3 months; (4) older than 18 years. The exclusion criteria were as follows: (1) with other carcinomas; (2) history of smoking and drinking; (3) with other intestinal diseases (e.g., inflammatory bowel disease, irritable bowel syndrome, or intestinal infection); (4) spouses do not eat or live together; (5) diabetes mellitus or hypertension; and (6) diet control. Stool specimens were obtained from each participant in the morning using a standard sample box and transported to the laboratory with an ice pack within 12 h. All the samples were frozen immediately and stored at −80 °C before examination. The study was approved by the Ethics Committee of the Seventh Medical Center of Chinese PLA General Hospital (no. 2020–090) and registered at chictr.org.cn (no. ChiCTR-DDD-17011169).

### DNA extraction

DNA was extracted from each fecal sample using a QIAamp Fast DNA Stool Mini Kit (Qiagen, Germany) per the manufacturer’s directions with some modifications. 1 ml of InhibitEX Buffer and the appropriate amount of glass beads (0.5 mm diameter, Qiagen) were added to each 200 mg of feces. A homogeneous instrument (FASTPREP-24, Aosheng Biotech, China) was used to homogenize the mixture at 60 Hz for 1 min twice. Afterward, DNA purification was conducted following the manufacturer’s instructions.

### 16S rRNA gene amplicon sequencing

Polymerase chain reaction (PCR; 95 °C for 3 min, followed by 30 cycles at 98 °C for 20 s, 58 °C for 15 s, and 72 °C for 20 s and a final extension at 72 °C for 5 min) was performed using the barcoded primers 341F 5’-CCTACGGGRSGCAGCAG-3’ and 806R 5’-GGACTACVVGGGTATCTAATC-3’ to amplify the V3-V4 region of the 16S ribosomal RNA genes. PCR was conducted using 2 × KAPA Library Amplification ReadyMix, primer, template DNA, and ddH_2_O. All 72 samples were amplified using the same procedures and reagents.

Amplicons were extracted from 2% agarose gels, purified with an AxyPrep DNA Gel Extraction Kit (Axygen Biosciences, Union City, CA, U.S.) per the manufacturer’s instructions, and quantified utilizing a Qubit® 2.0 (Invitrogen, U.S.). All quantified amplicons were mixed to obtain equal concentrations for sequencing using the Illumina MiSeq/HiSeq platform (Illumina, Inc., CA, USA). PANDAseq (https://github.com/ neufeld/pandaseq, version 2.9) was applied to overlap the 250 bp 3’ paired-end reads for concatenation into the original longer tags.

### Processing of sequencing data

QIIME 2 (version 2020.2) was used to analyze 16S rRNA gene amplicon sequencing data [17]. The sequencing data were subjected to quality control, amplicon sequence variants (ASVs) taxonomic assignment and contaminants removal. To evaluate alpha diversity, chao1, observed features and simpson diversity indices were used. For beta diversity analysis, the unweighted UniFrac distance metric was performed. The linear discrimination analysis (LDA) and effect size (LEfSe) analysis were performed to identify biomarkers that differed significantly between the two groups.

### Statistical analysis

Statistical analysis was performed using the R package and SPSS 26. Fisher’s exact test was applied to compare categorical data between groups. One-way ANOVA was used to compare multiple groups, followed by Dunnett’s test for comparison between two groups [18]. The significance level for all tests was set at 0.05 (two-tailed).

## Results

### Cohort description

**Fig. 1:**
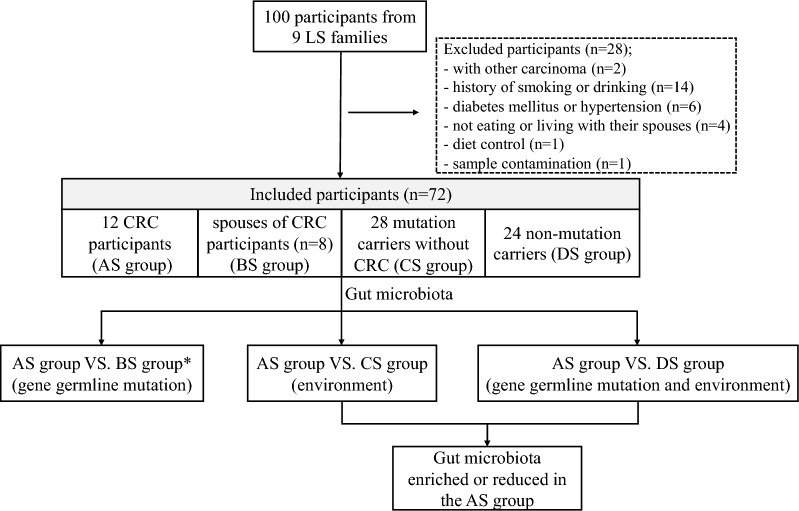
The flow chart of this study. *In this cohort, we analyzed the gut microbiota of only 8 patients in the AS group whose spouses were included in the BS group

A total of 100 participants were initially recruited in this study, but 28 participants were finally excluded, including 2 subjects with other carcinomas, 14 with a history of smoking or drinking, 6 with diabetes mellitus or hypertension, 4 who lived apart from their spouses, 1 with diet control and 1 with sample contamination. Finally, 72 participants were included in this study (Fig. 1). We examined and compared the fecal samples of 72 participants (33 males and 39 females), including 12 with LS-related CRC (AS group), their spouses (n = 8, BS group), 28 mutation carriers without CRC (CS group), and 24 non-mutation carriers (DS group). The mean age of these participants was 45.25 years in the AS group, 47.75 years in the BS group, 43.86 years in the CS group, and 37.63 years in the DS group. No apparent age or gender differences existed among the four groups (*F* = 1.824, *P* = 0.151; Fisher’s exact test = 0.662, *P* = 0.891, respectively). In the AS group, 7 patients had MLH1 mutations, 2 had PMS1 mutations, and 3 had MSH2 mutations. In the CS group, 6 participants were MLH1 mutation carriers, 4 were PMS1 mutation carriers, and 18 were MSH2 mutation carriers (Table 1 and Supplementary Table 1). The AS, BS, CS and DS groups shared the same top 10 phyla, but the relative abundance of phyla differed, as shown in the barplot (Supplementary Fig. 1).

**Table 1:** The basic characteristics of each group

Characteristic	Category	AS Group	BS Group	CS Group	DS Group
Number		12	8	28	24
Age (year)		45.25 ± 10.78	47.75 ± 8.73	43.86 ± 14.58	37.63 ± 13.26
Gender, n	Female	6	5	16	12
	Male	6	3	12	12
MMR mutation sites, n					
	MLH1	7	/	6	/
	PMS1	2	/	4	/
	MSH2	3	/	18	/

Data are presented as mean and SD

### Differences in the gut microbiome between the AS and BS groups

**Fig. 2:**
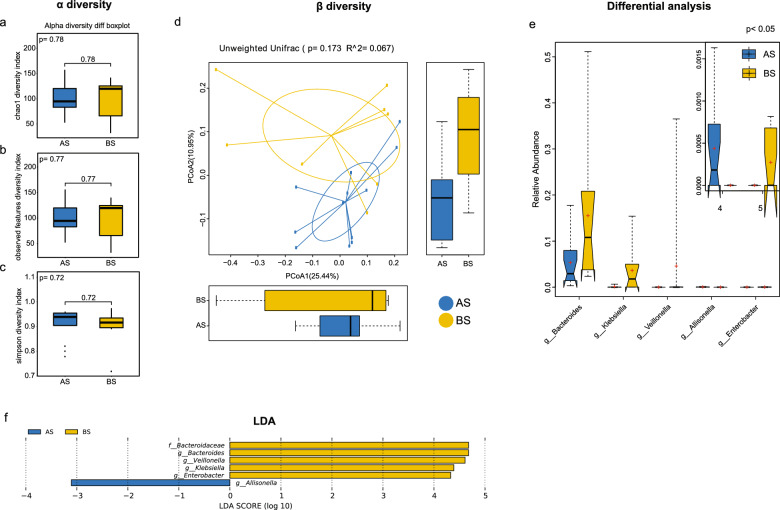
Gut microbiota analysis between Lynch Syndrome-related colorectal cancer patients (AS group) and their spouses (BS group). (**a**-**c**) The chao1 diversity, observed features diversity, and simpson diversity indices displayed the alpha diversity in the AS and BS groups. (**d**) Beta diversity of gut microbiota in the AS and BS groups using principal coordinate analysis (PCoA). (**e**) Differential gut microbiota between the AS and BS groups. (**f**) LEfSe analysis between the AS and BS groups. *P* < 0.05 indicated statistically significant

16S rRNA diversity analysis was conducted to investigate gut microbiome differences between LS-related CRC patients (AS group) and their spouses (BS group). The alpha diversity indices, including the chao1 diversity index, observed features diversity index, and simpson diversity index, exhibited no significant differences between the AS and BS groups (all *P* > 0.05, Fig. 2a-c). Beta diversity analysis of the variance in microbial communities between samples, measured by the unweighted UniFrac distance metric, also revealed no marked difference between the AS and BS groups (*P* = 0.173, Fig. 2d). Differential genera between the AS and BS groups were modest and negligible. Only *Allisonella* (*P* < 0.05) was enriched in the AS group, while *Bacteroides, Klebsiella, Veillonella and Enterobacter* (*P* < 0.05) levels were elevated in the BS group (Fig. 2e). LEfSe analysis revealed that the abundance of *Bacteroidaceae*, *Bacteroides*, *Veillonella*, *Klebsiella and Enterobacter* increased in the BS group, while only *Allisonella* increased in the AS group (LDA score > 3) (Fig. 2f). Moreover, no-phylum level difference was found between the AS and BS groups according to the LDA cladogram (Supplementary Fig. 2).

### Differences in the gut microbiome between the AS and CS groups

**Fig. 3:**
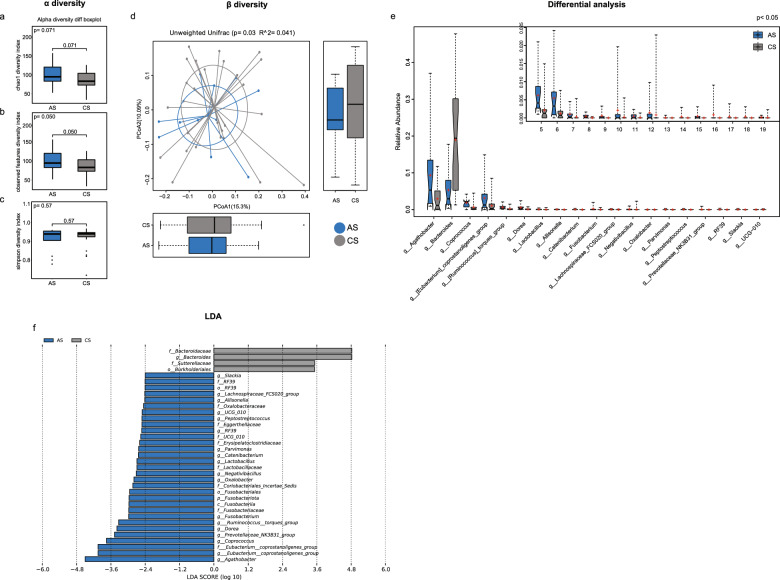
Gut microbiota analysis between Lynch Syndrome-related colorectal cancer patients (AS group) and mutation carriers without CRC (CS group). (**a**-**c**) The chao1 diversity, observed features diversity and simpson diversity indices in the AS and CS groups. (**d**) Beta diversity was analyzed by principal coordinate analysis (PCoA) in the AS and CS groups. (**e**) Differential microbiota between the AS and CS groups. (**f**) LEfSe analysis between the AS and CS groups. *P* < 0.05 indicated statistically significant

16S rRNA diversity analysis was performed to explore the difference in the gut microbiome between LS-related CRC patients (AS group) and mutation carriers without CRC (CS group). The alpha diversity indices between AS group and BS group were not significant, as validated by chao1 diversity index (*P* = 0.071), observed features diversity index (*P* = 0.050) and the simpson diversity index (*P* = 0.57) (Fig. 3a-c). However, Beta diversity analysis performed using the unweighted UniFrac distance metric revealed a significant difference between the AS and CS groups (*P* = 0.03, Fig. 3d).

The differences in the gut microbiota between the AS and CS groups were greater than those between the AS and BS groups. In detail, the abundance of *Agathobacter*, *Bacteroides*, *Coprococcus*, *Eubacterium_coprostanoligenes_group*, *Ruminococcus_torques_group*, *Dorea*, *Lactobacillus*, *Allisonella*, *Catenibacterium*, *Fusobacterium*, *Lachnospiraceae_FCS020_group*, *Negativibacillus*, *Oxalobacter*, *Parvimonas*, *Peptostreptococcus*, *Prevotellaceae_NK3B31_group*, *RF39*, *Slackia* and *UCG − 010* differed significantly between the AS and CS groups (*P* < 0.05, Fig. 3e). LEfSe analysis also confirmed that many gut microbes exhibited differences between the AS and CS groups. *Bacteroidaceae*, *Bacteroides*, *Sutterellaceae* and *Burkholderiales* were prevalent in the CS group, whereas *Slackia*, *RF39*, *Lachnospiraceae_FCS020_group*, *Allisonella*, *Oxalobacteraceae*, *UCG − 010*, *Peptostreptococcus*, *Eggerthellaceae*, *Ervsipelatoclostridiaceae*, *Parvimonas*, *Catenibacterium*, *Lactobacillus*, *Lactobacillaceae*, *Negativibacillus*, *Oxalobacter, Coriobacterials_Incertae_Sedis*, *Fusobacteriales*, *Fusobacteriota*, *Fusobacteria*, *Fusobacteriaceae*, *Fusobacterium*, *Ruminococcus_torques_group*, *Dorea*, *Prevotellaceae_NK3B31_group*, *Coprococcus*, *Eubacterium_coprostanoligenes_ group and Agathobacter* levels were elevated in the AS group (all LDA score > 2) (Fig. 3f). At the phylum level, *Fusobacteriota* was considered as a biomarker distinguishing the AS and CS groups (Supplementary Fig. 3).

### Differences in the gut microbiome between the AS and DS groups

**Fig. 4:**
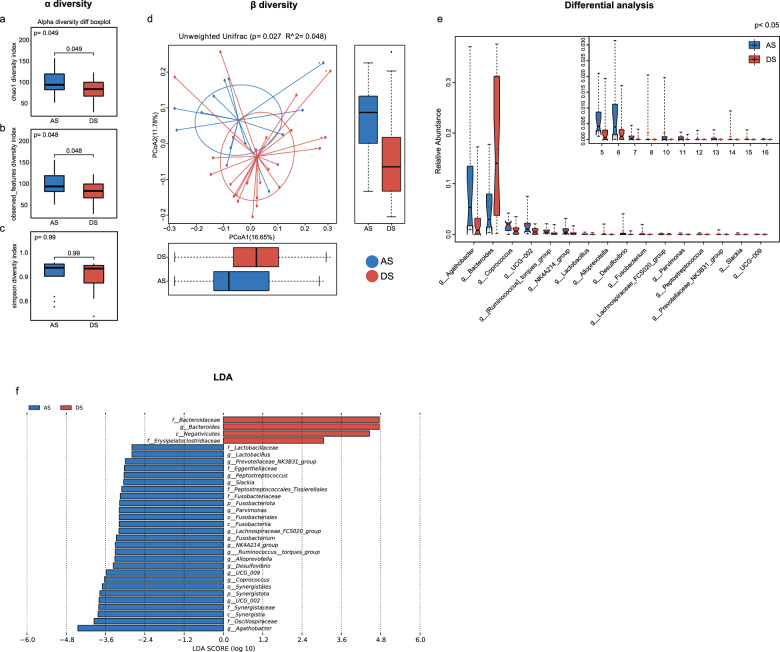
Gut microbiota analysis between Lynch Syndrome-related colorectal cancer patients (AS group) and non-mutation carriers (DS group). (**a**-**c**) The chao1 diversity, observed features diversity and simpson diversity indices in the AS and DS groups. (**d**) Beta diversity was analyzed by principal coordinate analysis (PCoA) in the AS and DS groups. (**e**) Differential microbiota between the AS and CS groups. (**f**) LEfSe analysis between the AS and DS groups. *P* < 0.05 indicated statistically significant

16S rRNA diversity analysis was conducted to assess the difference in the gut microbiome between LS-related CRC patients (AS group) and non-mutation carriers (DS group). The chao1 diversity index and the observed features diversity index, two alpha diversity indices, suggested a significant difference between the AS and DS groups (*P* = 0.049 and 0.048, respectively), while the simpson diversity index suggested no significant difference between the AS and DS groups (*P* > 0.05, Fig. 4a-c). Beta diversity analysis, assessed by the unweighted UniFrac distance metric, revealed a significant difference between the AS and DS groups (*P* = 0.027, Fig. 4d). In detail, *Agathobacter*, *Bacteroides*, *Coprococcus*, *UCG − 002*, *Ruminococcus_torques_group*, *NK4A214_group*, *Lactobacillus*, *Alloprevotella*, *Desulfovibrio*, *Fusobacterium*, *Lachnospiraceae_FCS020_group*, *Parvimonas*, *Peptostreptococcus*, *Prevotellaceae_NK3B31_group*, *Slackia* and *UCG − 009* levels differed significantly between the AS and DS groups (all *P* < 0.05, Fig. 4e). LEfSe analysis found that *Bacteroidaceae*, *Bacteroides*, *Negativicutes*, *and Erysipelatoclostridiaceae* elevated in the DS group, while *Lactobacillaceae*, *Lactobacillus*, *Prevotellaceae_NK3B31_ group*, *Eggerthellaceae*, *Peptostreptococcus*, *Slackia*, *Peptostreptococcales_ Tissierellales*, *Fusobacteriaceae*, *Fusobacteriota*, *Parvimonas*, *Fusobacteriales*, *Fusobacteriia*, *Lachnospiraceae_FCS020_group*, *Fusobacterium*, *NK4A214_group*, *Ruminococcus_torques_group*, *Alloprevotella*, *Desulfovibrio*, *UCG − 009*, *Coprococcus*, Synergistales, Synergistota, *UCG − 002, Synergistaceae*, *Synergistia*, *Oscillospiraceae and Agathobacter* appeared to be enriched in the AS group (all with LDA scores > 2) (Fig. 4f). *Fusobacteriota* and *Synergistota* were considered as phylum-level markers between the AS and DS groups (Supplementary Fig. 4).

### Microbe genera involvement in the development of CRC in LS families

Combining the results of the AS group vs. CS group and the AS group vs. DS group comparisons, we identified ten microbe genera enriched in the AS group, including *Agathobacter*, *Coprococcus*, *Ruminococcus_torques_group*, *Lactobacillus*, *Lachnospiraceae_FCS020_group*, *Fusobacterium*, *Parvimonas*, *Peptostreptococcus*, *Prevotellaceae_NK3B31_group* and *Slackia*. A reduction in *Bacteroides* was also found in the AS group.

## Discussion

Diet serves as an important risk factor for CRC [19], and can alter the composition of gut microbiota [20]. Thus, we enrolled the spouse of LS-related CRC patient in the BS group to reduce the impact of environmental factors. This study revealed no marked difference in alpha or beta diversity between the AS and BS groups. Additionally, the relative abundance of differential bacteria between the AS and BS groups was small and negligible. *Allisonella* was found to be more abundant in the AS group, whereas *Bacteroides*, *Klebsiella*, *Veillonella* and *Enterobacter* were more prevalent in the BS group. It is noteworthy that the relative abundance of *Allisonella*, *Veillonella* and *Enterobacter* was low. These findings confirmed previous studies by demonstrating the major role of germline mutations in tumorigenesis, with gut microbiota playing a minor role.

Few studies have been conducted on the role of *Allisonella* in CRC. However, several studies have demonstrated the relationship between *Allisonella* and disease. *Allisonella* was found to be markedly enhanced in patients with chronic radiation-related proctitis caused by pelvic cancer radiotherapy [21]. Furthermore, *Allisonella* was enriched in hepatitis B virus-related hepatocellular carcinoma patients with post-hepatectomy liver failure compared to those without post-hepatectomy liver failure [22]. *Bacteroides* have long been reported to be closely associated with CRC [23]. However, we noticed that *Bacteroides* was reduced in the AS group, which may be attributed to the compensatory effects or differences in the etiology of CRC. The presence of *Klebsiella* may be related to the absence of CRC. *Klebsiella* proportions were higher in post-surgery CRC patients than in non-surgery CRC patients [24]. Treatment with oxaliplatin and capecitabine increased *Veillonella* levels in postoperative CRC patients [25], which coincides with the protective effect of *Veillonella* in this study. A decreased amount of *Enterobacter* may contribute to high intestinal susceptibility to carcinogenic factors in high-fat and high-calorie diet Wistar rats [26]. It is worth investigating whether a reduction in *Enterobacter* abundance increases gut susceptibility to carcinogens in LS families.

Gut microbiota has been reported to induce carcinogenesis in MSH2-deficient mice [27]. To further investigate the involvement of gut microbiota in the development of CRC in LS families, we investigated and evaluated the gut microbiota compositions between the AS and CS groups and identified significant differences between the two groups. MMR mutations were found in both the AS and CS groups. Thus, for people with MMR mutations, the gut microbiota plays a critical role in the development of CRC in LS families. A total of 19 biomarkers at the genus level were detected between the AS and CS groups, providing new insights into the prevention and management of CRC in LS families. The AS group and DS group have different genetic mutations and living environments, and exhibited more pronounced differences in the gut microbiota (greater alpha and beta diversity differences), indicating that genetic germline mutation and environment both influence the development of CRC in LS families. We observed 16 differential genera in this cohort.

Combining the results of the comparison between the AS and CS groups and the AS and DS groups, we identified ten microbial genera enriched in the AS group, namely, *Agathobacter*, *Coprococcus*, *Ruminococcus_torques_group*, *Lactobacillus*, *Lachnospiraceae_FCS020_group*, *Fusobacterium*, *Parvimonas*, *Peptostreptococcus*, *Prevotellaceae_NK3B31_group* and *Slackia*. A decreased abundance of *Bacteroides* in the AS group was also identified. *Agathobacter*, a butyrate-producing genus [28], was shown to be more prevalent in healthy individuals than in CRC patients [29], and its presence was more closely related to the absence of lymph node metastasis in CRC [30]. *Coprococcus*, functioning as a butyrate producer, suppresses cancer development by modulating Akt/ERK, Wnt, and TGF-β signaling [31]. *Prevotellaceae_NK3B31_group* is capable of producing butyrate [32] and acts as a protector of the intestinal mucosa [33]. However, in this study, *Agathobacter*, *Coprococcus* and *Prevotellaceae_NK3B31_group* were enriched in the AS group. Butyrate has long been considered a protective factor of CRC. Despite this, the adverse effect of butyrate in CRC has also been reported. Gavaging *Apc*^*Δ14/*+^mice with butyrate-producing bacteria (*Porphyromonas gingivalis* and *Porphyromonas asaccharolytica*) increased the numbers of colorectal tumors compared to PBS mice, and supplementing with butyrate synthesis defective mutant of *Porphyromonas gingivalis* significantly reduced the numbers of colorectal tumors compared to wild-type *Porphyromonas gingivalis* [34]. Likewise, in a randomized control study supplementing LS patients with resistance starch or placebo increased concentrations of butyrate in the colon but no differences in CRC development were detected between the two groups [35]. Thus, the role of these elevated butyrate producing bacteria in LS-related CRC needs further research. The elevated level of *Coprococcus* in CRC may be explained by MMR status. One study enrolled 45 sporadic CRC patients and discovered that the abundance of *Coprococcus* spp. in the subgroup with MLH1 defects was considerably greater than that in the subgroup with intact MLH1 [36]. In this study, we recruited patients with MLH1, MSH2 and PMS1 mutations, all of which shared an identical genetic factor, and revealed that *Coprococcus* was elevated in LS-related CRC patients with MMR deficiency.

*Lactobacillus* was perceived to have a health-promoting property [37]. A systematic review of 31 studies indicated that the abundance of *Lactobacillus* was lower in faecal samples of CRC compared to healthy controls, but this difference was not statistically significant [38]. *Lachnospiraceae_FCS020_group* (*OR* = 0.607) preserves as a protective factor against CRC [39]. However, in this study, both *Lactobacillus* and *Lachnospiraceae_FCS020_group* were elevated in the AS group, which might be interpreted as body compensation, different inclusion and exclusion criteria, or a difference in CRC etiology. *Lactobacillus* metabolizes tryptophan into indole-3-lactic acid (ILA) which boots indole-3-propionic acid (IPA) and indole-3-acetic acid (IAA) synthesis via microbial cross-feeding [40]. ILA, IPA and IAA have been proved to alleviate intestinal inflammation and modify gut microbiota in both DSS-induced and IL-10^−/−^ spontaneous colitis models [40]. *Lactobacillus* might possess a similar protective effect in LS-related CRC which requires further research. It has been reported that smoking-induced dysbiosis of gut microbiota promotes CRC by activating oncogenic MAPK/ERK signaling in the colonic epithelium [41]. To obtain plausible results, we excluded smokers from this study. Furthermore, the participants in this study come from six different China provinces, reducing regional disparities to some extent.

Intestinal polyps develop years prior to CRC. A previous study disclosed that the abundance of *Ruminococcus_torques_group* in both the hyperplastic polyps and conventional adenomas was markedly greater than in healthy controls [42]. In a study examining 95 paired specimens of CRC and normal colonic DNA, 16S rDNA sequence analysis indicated that *Fusobacterium* was enriched in CRC [43]. Additionally, patients with invasive CRC had significantly higher *Fusobacterium* levels than those with early CRC [44]. A substantial enhancement of *Parvimonas* was also observed in the stool of CRC patients, and *Fusobacterium* in intestinal tissue has been demonstrated positively correlated with fecal *Parvimonas* [45]. *Peptostreptococcus* were significantly increased in CRC patients compared with healthy controls [46]. *Slackia* has been shown to be positively related to CRC risk [47], and an increased abundance of *Slackia* is associated with shorter disease-free survival rates [48]. The results presented above were consistent with our findings, indicating that the elevated abundance of *Ruminococcus_torques_group*, *Fusobacterium*, *Parvimonas*, *Peptostreptococcus* and *Slackia* may be related to the development of CRC.

*Bacteroides* has been linked to an increased risk of CRC [23]. The abundance of *Bacteroides* in patients with metastatic CRC was significantly higher than in the non-metastatic CRC [49]. As shown in the animal model, *Bacteroides* occupied a relatively higher abundance in 1, 2-dimethylhydrazine-induced CRC rats compared to controls [50]. In this study, the abundance of *Bacteroides* was lower in the LS-related CRC group than in the non-CRC group. The decrease in *Bacteroides* may be protective, other microbiota may contribute to the development of CRC in LS families.

This study analyzed the role of gut microbiota and genetic factors in the development of CRC in LS families. However, we did not analyze the function of these differential microbial genera, and more research is required to determine the underlying mechanism.

## Conclusion

This study emphasized the role of gut microbiota in the development of LS-related CRC apart from germline gene mutations. We identified three butyrate-producing genera elevated in LS-related CRC, and the underlying mechanism warrants further research. Careful probiotics or antibiotics intervention might be promising strategies for preventing and managing CRC in LS families.

## Supplementary Information

Additional file 1.

Additional file 2.

## Data Availability

All data supporting the findings of this study are available within the article and its Supplemental Information files. The data generated and analyzed during the present study are available from the corresponding author upon reasonable request.

## Abbreviations

ASVs: Amplicon sequence variants
CRC: Colorectal cancer
LS: Lynch syndrome
LDA: Linear discrimination analysis
LEfSe: LDA effect size
MMR: Mismatch repair
