# Supplementary material for: Colorectal cancer in Lynch syndrome families: consequences of gene germline mutations and the gut microbiota
Source: Orphanet J Rare Dis. 2025 Jan 18;20:30. doi: 10.1186/s13023-025-03543-4 (PMC11742751; doi:10.1186/s13023-025-03543-4)
Supplement: Supplementary file 1 — Additional file 1. [file 13023_2025_3543_MOESM1_ESM.docx]

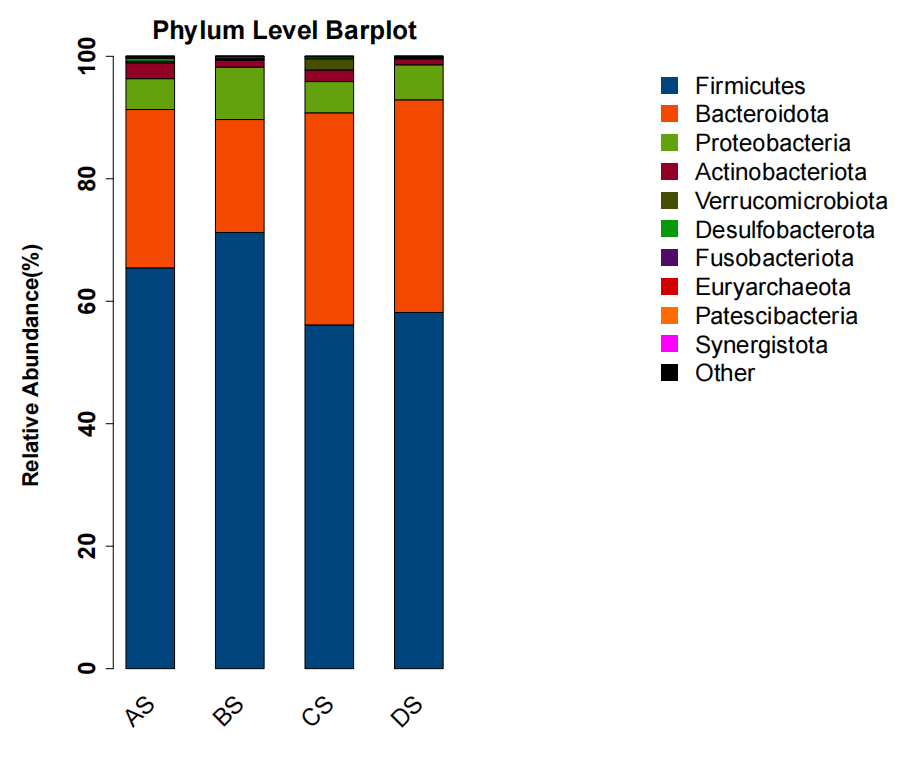


**Supplementary Fig. 1** The phylum level barplot for the AS, BS CS and DS groups.


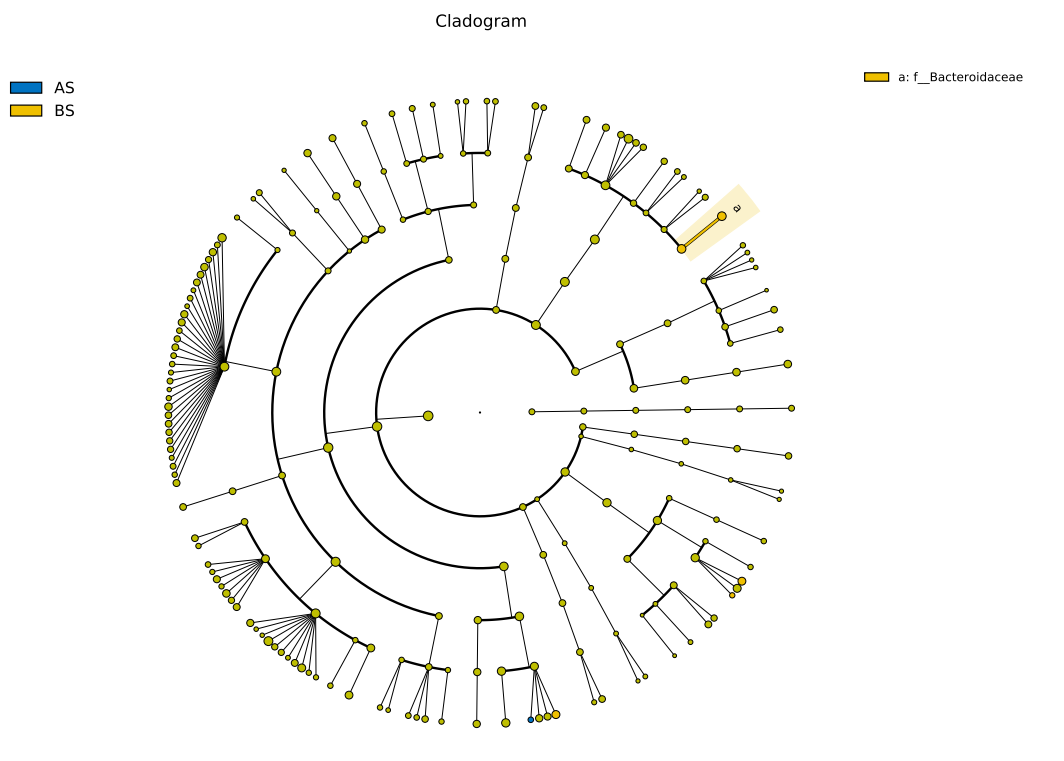


**Supplementary Fig. 2** The LDA Cladogram of the AS and BS groups. No phylum marker was identified between the AS and BS groups.


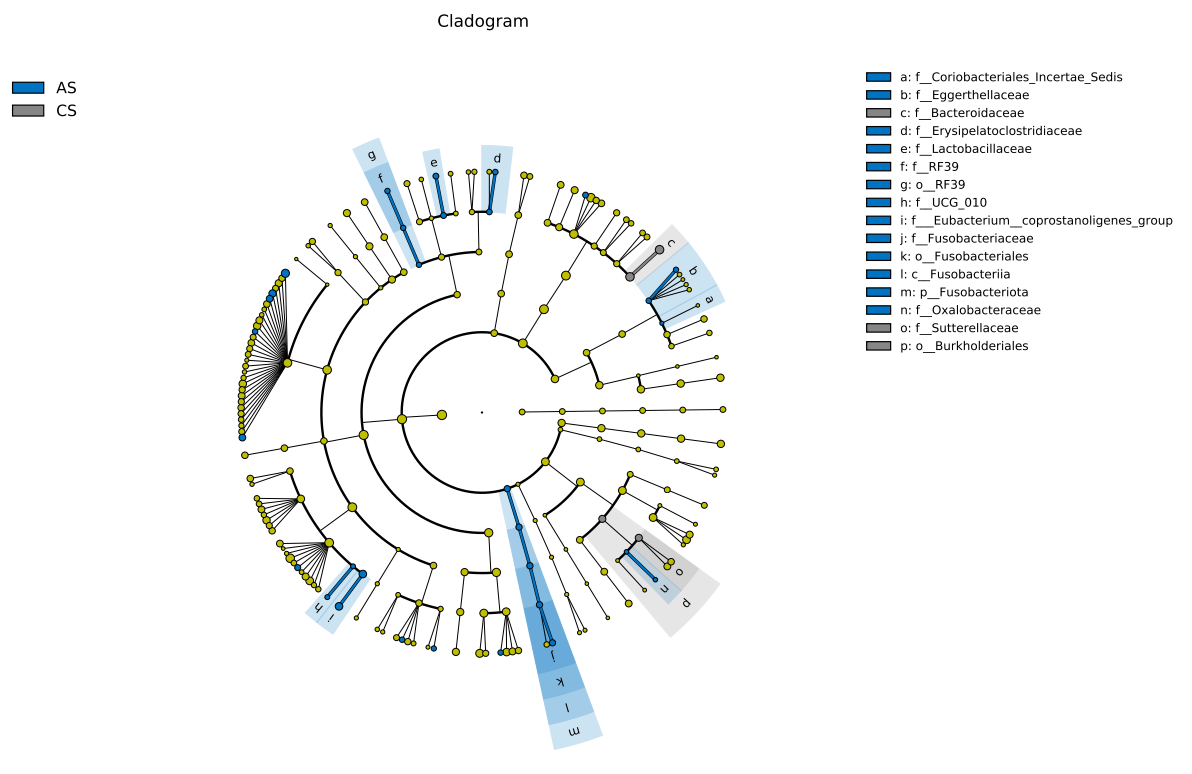


**Supplementary Fig. 3** The LDA Cladogram of the AS and CS groups. The AS and CS groups shared one phylum marker, p__Fusobacteriota (m: p__Fusobacteriota).


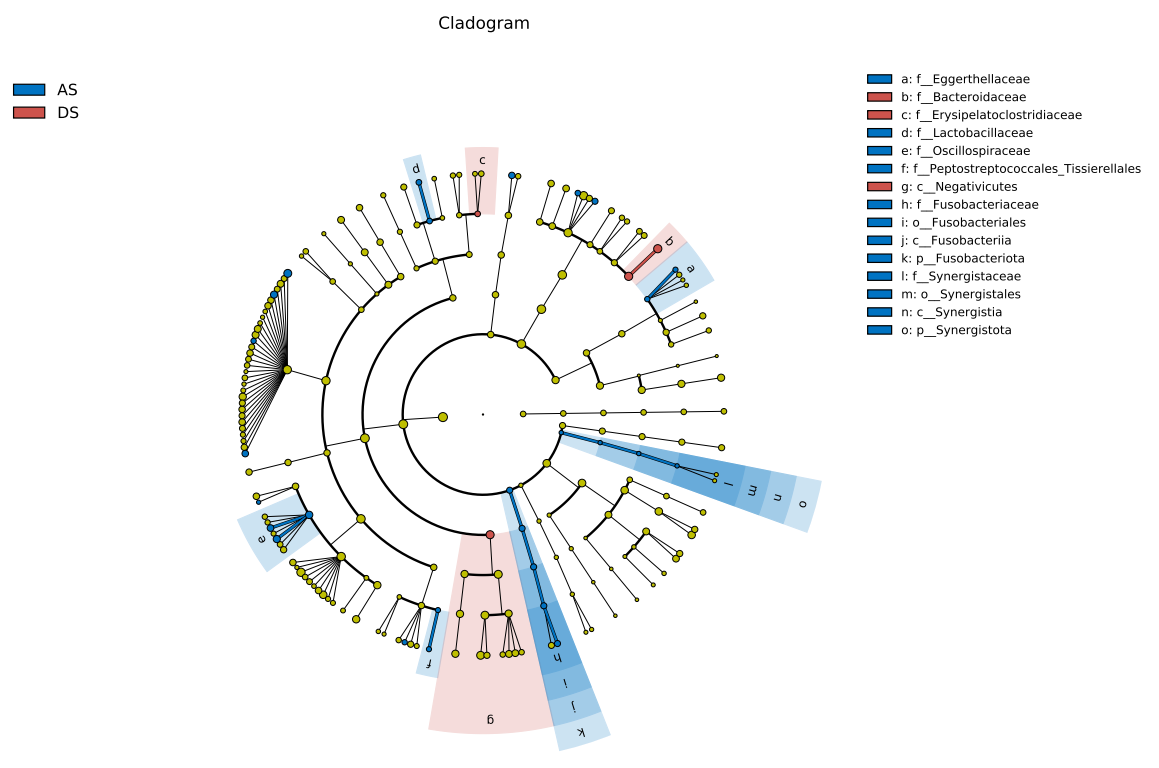


**Supplementary Fig. 4** The LDA Cladogram of the AS and DS groups. Two phylum markers, p__Fusobacteriota (k: p__Fusobacteriota) and p__Synergistota (o: p__Synergistota), were identified between the AS and DS groups.
