# Supplementary material for: Colorectal cancer in Lynch syndrome families: consequences of gene germline mutations and the gut microbiota
Source: Orphanet J Rare Dis. 2025 Jan 18;20:30. doi: 10.1186/s13023-025-03543-4 (PMC11742751; doi:10.1186/s13023-025-03543-4)
Supplement: Supplementary file 2 — Additional file 2. [file 13023_2025_3543_MOESM2_ESM.docx]

**Supplementary Table 1. The basic characteristics of all subjects.**

|  | **Group** | **Age (year)** | **Gender** | **MMR mutation sites and mutation types** |
| --- | --- | --- | --- | --- |
|  | AS-1 | 42 | Male | hMSH2 exon-13 IVS13-2 A>C |
|  | AS-2 | 50 | Male | hMLH1 exon-19 c.2141G>A |
|  | AS-3 | 28 | Female | hMLH1 exon-19 c.2141G>A |
|  | AS-5^*^ | 36 | Female | MLH1: NC_000003.11:g.37067499G>A |
|  | AS-6 | 57 | Male | MLH1:NM_001258271:exon6:c.498dupA:p.L166fs |
|  | AS-7 | 32 | Female | MLH1:NM_001258271:exon6:c.498dupA:p.L166fs |
|  | AS-8 | 53 | Male | PMS1 exon-5 c.402 G>C |
|  | AS-9 | 39 | Male | PMS1 exon-5 c.402 G>C |
|  | AS-10 | 47 | Female | MSH2:NM_000251:IVS12:c.2006-1:G>A |
|  | AS-11 | 57 | Male | hMSH2 exon-3 c.610G>T |
|  | AS-12 | 62 | Female | hMLH1 exon-11 c.910 T>A |
|  | AS-13 | 40 | Female | hMLH1 exon-2 c.199dupG |
|  | BS-1 | 41 | Female |  |
|  | BS-2 | 48 | Female |  |
|  | BS-3 | 39 | Male |  |
|  | BS-4 | 51 | Female |  |
|  | BS-5 | 37 | Female |  |
|  | BS-6 | 48 | Male |  |
|  | BS-7 | 55 | Female |  |
|  | BS-8 | 63 | Male |  |
|  | CS-1 | 38 | Female | hMSH2 exon-13 IVS13-2 A>C |
|  | CS-2 | 56 | Female | hMSH2 exon-13 IVS13-2 A>C |
|  | CS-3 | 46 | Male | hMSH2 exon-13 IVS13-2 A>C |
|  | CS-4 | 66 | Male | hMSH2 exon-13 IVS13-2 A>C |
|  | CS-5 | 59 | Female | hMSH2 exon-13 IVS13-2 A>C |
|  | CS-6 | 39 | Male | hMLH1 exon-19 c.2141G>A |
|  | CS-7 | 59 | Female | MLH1: NC_000003.11:g.37067499G>A |
|  | CS-8 | 30 | Male | MLH1:NM_001258271:exon6:c.498dupA:p.L166fs |
|  | CS-10 | 53 | Male | MLH1:NM_001258271:exon6:c.498dupA:p.L166fs |
|  | CS-11 | 32 | Female | MLH1:NM_001258271:exon6:c.498dupA:p.L166fs |
|  | CS-12 | 29 | Female | MLH1:NM_001258271:exon6:c.498dupA:p.L166fs |
|  | CS-13 | 32 | Female | PMS1 exon-5 c.402 G>C |
|  | CS-14 | 57 | Female | PMS1 exon-5 c.402 G>C |
|  | CS-15 | 55 | Male | PMS1 exon-5 c.402 G>C |
|  | CS-16 | 37 | Male | PMS1 exon-5 c.402 G>C |
|  | CS-17 | 70 | Female | MSH2:NM_000251:IVS12:c.2006-1:G>A |
|  | CS-18 | 45 | Female | MSH2:NM_000251:IVS12:c.2006-1:G>A |
|  | CS-19 | 44 | Male | MSH2:NM_000251:IVS12:c.2006-1:G>A |
|  | CS-20 | 21 | Female | MSH2:NM_000251:IVS12:c.2006-1:G>A |
|  | CS-21 | 15 | Male | MSH2:NM_000251:IVS12:c.2006-1:G>A |
|  | CS-22 | 66 | Female | hMSH2 exon-3 c.610G>T |
|  | CS-23 | 64 | Male | hMSH2 exon-3 c.610G>T |
|  | CS-24 | 35 | Male | hMSH2 exon-3 c.610G>T |
|  | CS-25 | 35 | Male | hMSH2 exon-3 c.610G>T |
|  | CS-26 | 31 | Female | hMSH2 exon-3 c.610G>T |
|  | CS-27 | 31 | Female | hMSH2 exon-3 c.610G>T |
|  | CS-28 | 45 | Female | hMSH2 exon-3 c.610G>T |
|  | CS-29 | 38 | Female | hMSH2 exon-3 c.610G>T |
|  | DS-1 | 41 | Female |  |
|  | DS-2 | 44 | Male |  |
|  | DS-3 | 42 | Female |  |
|  | DS-4 | 26 | Male |  |
|  | DS-5 | 41 | Female |  |
|  | DS-6 | 39 | Female |  |
|  | DS-7 | 69 | Male |  |
|  | DS-8 | 56 | Female |  |
|  | DS-9 | 47 | Male |  |
|  | DS-10 | 53 | Male |  |
|  | DS-11 | 21 | Female |  |
|  | DS-12 | 51 | Female |  |
|  | DS-13 | 28 | Male |  |
|  | DS-14 | 22 | Male |  |
|  | DS-15 | 29 | Female |  |
|  | DS-16 | 36 | Female |  |
|  | DS-17 | 46 | Female |  |
|  | DS-18 | 22 | Female |  |
|  | DS-19 | 54 | Male |  |
|  | DS-20 | 27 | Male |  |
|  | DS-21 | 16 | Male |  |
|  | DS-22 | 32 | Male |  |
|  | DS-23 | 33 | Male |  |
|  | DS-24 | 28 | Female |  |
